# Antibiotic Resistance in Enterococci and *Enterobacteriaceae* from Laboratory-Reared Fresh Mealworm Larvae (*Tenebrio molitor* L.) and Their Frass

**DOI:** 10.3390/pathogens13060456

**Published:** 2024-05-28

**Authors:** Barbara Turchi, Simone Mancini, Francesca Pedonese, Ilaria Resci, Beatrice Torracca, Francesca Marconi, Chiara Barone, Roberta Nuvoloni, Filippo Fratini

**Affiliations:** 1 Department of Veterinary Sciences, University of Pisa, Viale delle Piagge 2, 56124 Pisa, Italy; barbara.turchi@unipi.it (B.T.); simone.mancini@unipi.it (S.M.); ilaria.resci@phd.unipi.it (I.R.); beatrice.torracca@unipi.it (B.T.); francesca.marconi@phd.unipi.it (F.M.); chiara.barone91@gmail.com (C.B.); roberta.nuvoloni@unipi.it (R.N.); filippo.fratini@unipi.it (F.F.); 2 Interdepartmental Center ‘NUTRAFOOD’, University of Pisa, Via del Borghetto 80, 56124 Pisa, Italy

**Keywords:** antibiotic susceptibility, edible insects, *Enterobacteriaceae*, *Enterococcus* spp.

## Abstract

The occurrence of antibiotic-resistant bacteria in foodstuff involves a human health risk. Edible insects are a precious resource; however, their consumption raises food safety issues. In this study, the occurrence of antibiotic resistant bacteria in laboratory-reared fresh mealworm larvae (*Tenebrio molitor* L.) and frass was assessed. Antibiotics were not used during the rearing. *Enterobacteriaceae* and enterococci were isolated from 17 larvae and eight frass samples. In total, 62 and 69 isolates presumed to belong to *Enterobacteriaceae* and *Enterococcus* spp., respectively, were obtained and tested for antibiotic susceptibility via disk diffusion. Based on the results, isolates were grouped, and representative resistant isolates were identified at species level through *16S rRNA* gene sequencing. For enterococci resistance, percentages higher than 15% were observed for vancomycin and quinupristin–dalfopristin, whereas *Enterobacteriaceae* resistance higher than 25% was found against cefoxitin, ampicillin, and amoxicillin–clavulanic acid. Based on the species identification, the observed resistances seemed to be intrinsic both for enterococci and *Enterobacteriaceae*, except for some β-lactams resistance in *Shigella boydii* (cefoxitin and aztreonam). These could be due to transferable genetic elements. This study suggests the need for further investigations to clarify the role of edible insects in the spreading of antibiotic resistance determinants through the food chain.

## 1. Introduction

The Food and Agriculture Organization of the United Nations (FAO) estimates that in 2050 the world population will reach the 9 billion mark, with a consequent growth in the need for food of animal origin and raw materials for food and feed production [1]. To meet the increase in food consumption, it is estimated that meat and dairy production will have to be doubled, while to meet the global need for proteins, fish production from aquaculture will even have to be tripled [2].

Indeed, there is no doubt that foods of animal origin represent the main sources of proteins of high biological value. In this context, the most challenging aspect for agribusinesses is the production of animal proteins in high quantities, with a lower environmental impact than that of intensive food production systems, and with an optimal nutritional and hygienic profile [3]. Currently, insects represent one of the most promising alternatives as a new protein source and the FAO has been promoting the breeding of insects for human and animal consumption [4,5].

Indeed, the European Food Safety Authority (EFSA) recently released several positive opinions favoring the use of different insect species in approved novel food and the European Union (EU) authorized the use of several insects species as novel food and in feed for fish, poultry, and swine as well as in pet food [5,6]. Among the approved species, *Tenebrio molitor* is considered one of best candidates for mass production from a food and feed security perspective due to its nutritional value and high feed conversion rate [2,7]. Moreover, *T. molitor* is one of the most acceptable insect species for consumers [8,9].

Among the main obstacles to the trade and use of edible insects, we must consider the limited knowledge regarding their possible role as vehicles of chemical and microbiological hazards [10]. Recent studies have characterized the bacterial communities of the main edible insects’ gut microbiota; however, available data are not yet sufficient [11,12]. Other studies have investigated the potential persistence of different food pathogens, such as *Listeria monocytogenes*, *Staphylococcus aureus*, and *Salmonella enterica* in the insects’ rearing systems [13,14,15]. According to the One Health concept, the health of humans, animals, plants, and their shared environment are closely interconnected and the spread of antimicrobial resistant microorganisms through the food and feed supply is a well-known issue requiring a One Health approach [16]. In this regard, the number of studies concerning the possible role of edible insects in the transmission of genes coding for antibiotic resistance is still low [17,18,19,20]. The phenomenon of antibiotic resistance was recognized in the 1950s, but in the past decade it has experienced a vertical upsurge, becoming a global problem affecting both developed and developing countries. Genetic determinants responsible for this phenomenon are easily exchanged through horizontal transmission among different bacterial communities. It is known that insects are characterized by high bacterial loads both on the outer integument and in the gut [21]. The role of these insect-associated microorganisms in spreading antibiotic resistance genes is a concern that needs to be further investigated [22].

The aim of this work was to isolate, identify, and evaluate the susceptibility to antibiotics commonly used in human and veterinary medicine of isolates belonging to the *Enterobacteriaceae* family and *Enterococcus* spp. from edible insect larvae of *T. molitor* species and samples of frass.

## 2. Materials and Methods

### 2.1. Sampling and Bacteria Isolation

In total, 25 samples were analyzed; 17 of them were represented by *T. molitor* larvae (10 g) and 8 by frass samples (10 g). *T. molitor* larvae were reared in laboratory conditions at the Department of Veterinary Sciences of the University of Pisa (Italy). The larvae were kept at a temperature of 25 ± 1 °C and a relative humidity of 50–60%. The insects were reared on unsold food or former foodstuff, such as spent brewery grains, and bread of different types (white, wholemeal, spicy, mixed). As a source of water supply, potato slices were administered once or twice a week depending on the stage of development of the larvae. Frass samples were obtained by fasting the larvae for 48 h in boxes with perforated bottom to avoid contact between the larvae and the frass. Frass was thus collected in a previously sterilized container located under the boxes. No antibiotics were administered during the entire rearing cycle. Each sample was placed inside a sterile tube, identified by a sequential number, and processed as follows: 10 g of larvae were taken and placed inside 50 mL conical Falcon centrifuge tubes into which an aliquot of sterile saline solution was added. Obtained samples were stirred for one minute to reduce external microbial contamination. The washing solution was then removed. Washed larvae were collected into a sterile Stomacher bag with 90 mL of sterile saline solution and then subjected to crushing and homogenization. The homogenate was collected with a sterile loop and transferred onto a Petri dish containing VRBGA medium (Violet Red Bile Glucose Agar, Thermo Fisher Scientific, Milan, Italy) for the isolation of *Enterobacteriaceae* or KAA (Kanamycin Aesculin Azide Agar, Thermo Fisher Scientific, Milan, Italy) for *Enterococcus* spp. VRBGA plates were incubated at 37 °C for 24 h, while KAA plates at 42 °C for 48 h. For frass samples, 10 g of substrate were placed in 50 mL conical Falcon centrifuge tubes with 50 mL of sterile saline solution; the sample was then mixed using a vortex mixer. The solution obtained was collected with a sterile loop and inoculated on the same cultural media reported above. After the incubation period, bacterial growth was assessed for both *Enterobacteriaceae* and *Enterococcus* spp. One to three typical colonies (according to the culture media manufacturer’s instructions) of *Enterobacteriaceae* and *Enterococcus* spp. from positive samples were subcultured to obtain pure isolates. Obtained isolates were inoculated in BHI broth (Brain Heart Infusion, Thermo Fisher Scientific, Milan, Italy) and incubated at 37 °C per 24 h. After incubation, broth cultures were had 15% glycerol (Microbiol Diagnostici, Cagliari, Italy) added and were stored at −20 °C. Isolates were then tested for their antibiotic resistance profile and for identification purposes.

### 2.2. Determination of Antibiotic Resistance Profile

The disk-diffusion antimicrobial susceptibility testing method was applied using a bacterial inoculum prepared in sterile saline adjusted to the density of a McFarland 0.5 standard (Remel™ McFarland Equivalence Turbidity Standards, Thermo Fisher Scientific, Milan, Italy). The inoculum was evenly spread over the entire surface of an MH agar plate (Mueller Hinton, Thermo Fisher Scientific, Milan, Italy) by swabbing. After the application of antimicrobial disks (Thermo Fisher Scientific, Milan, Italy), the plates were incubated at 37 °C for 24 h. After incubation, inhibition zones were read and zone diameters were interpreted and categorized as susceptible, intermediate, or resistant according to the CLSI [23] and EUCAST [24] breakpoint tables for *Enterobacterales* and enterococci. *Escherichia coli* ATCC 25922 and *Staphylococcus aureus* ATCC 25923 were used as control strains for *Enterobacteriaceae* and enterococci, respectively. The following antimicrobials were tested for *Enterobacteriaceae*: ampicillin 10 µg, amoxicillin–clavulanic acid 30 µg, cefoxitin 30 µg, cefotaxime 30 µg, chloramphenicol 10 µg, tetracycline 30 µg, trimethoprim–sulfamethoxazole 25 µg, gentamicin 30 µg, streptomycin 10 µg, imipenem 10 µg, and aztreonam 30 µg, while the following antimicrobials were used for enterococci: tetracycline 30 µg, ciprofloxacin 5 µg, vancomycin 5 µg, imipenem 10 µg, erythromycin 15 µg, ampicillin 10 µg, gentamicin 120 µg, linezolid 30 µg, chloramphenicol 30 µg, and quinupristin–dalfopristin 15 µg. Specifically, for *Enterobacteriaceae*, CLSI breakpoints were applied for tetracycline, streptomycin, and cefotaxime, while for enterococci, CLSI breakpoints were applied for ampicillin, quinupristin–dalfopristin, tetracycline, erythromycin, ampicillin, gentamicin, linezolid, and chloramphenicol. The isolates were then grouped into clusters based on their phenotypic antibiotic susceptibility profile. Hierarchical clustering was performed with R studio version 4.2.1 using packages *factoextra*, *FactoMineR*, *ggplot2*, *Rcolorbrewer*, *ggpubr*, and *tidyverse*.

### 2.3. 16S rDNA Sequencing for Bacterial Isolates Identification

At least one isolate from each profile presenting at least one resistance to the antibiotics tested was identified. Identification at the species level was performed via *16S rDNA* gene sequencing according to [25]. The commercial kit “GenEluteTM Bacterial Genomic DNA kit” (Sigma-Aldrich, St. Louis, MO, USA) was used for DNA extraction following the protocol provided by the manufacturer. EconoTaq^®®^ PLUS 2X Master Mix (Lucigen, Madison, WI, USA) was used for the amplification of *16S rDNA* genes. The amplicons (1300 bp) were sent to BMR Genomics (Padua, Italy) for sequencing. The GenBank database was employed to attribute the species using the BLASTN local alignment search tool.

## 3. Results

### 3.1. Determination of Antibiotic Resistance Profile and Identification of Enterococcus

All the 25 analyzed samples (100%) were positive for the presence of bacteria presumably belonging to *Enterococcus* spp. Sixty-nine pure isolates were obtained. In total, 53.6% of them were susceptible to all antibiotics tested, while 29% showed an intermediate resistance to at least one tested antibiotic. Regarding resistance rates, 36.2% of isolates were resistant to at least one of the ten antibiotics included in this study. The highest resistance was found against vancomycin (20.3%), followed by quinupristin–dalfopristin (15.9%) (Table 1). No resistance was detected against tetracycline, erythromycin, ciprofloxacin, linezolid, ampicillin, imipenem, chloramphenicol, ciprofloxacin, or gentamicin. Intermediate resistance was found against quinupristin–dalfopristin (10.1%) and erythromycin (24.6%). No multiresistant isolates were identified.

**Figure 1:**
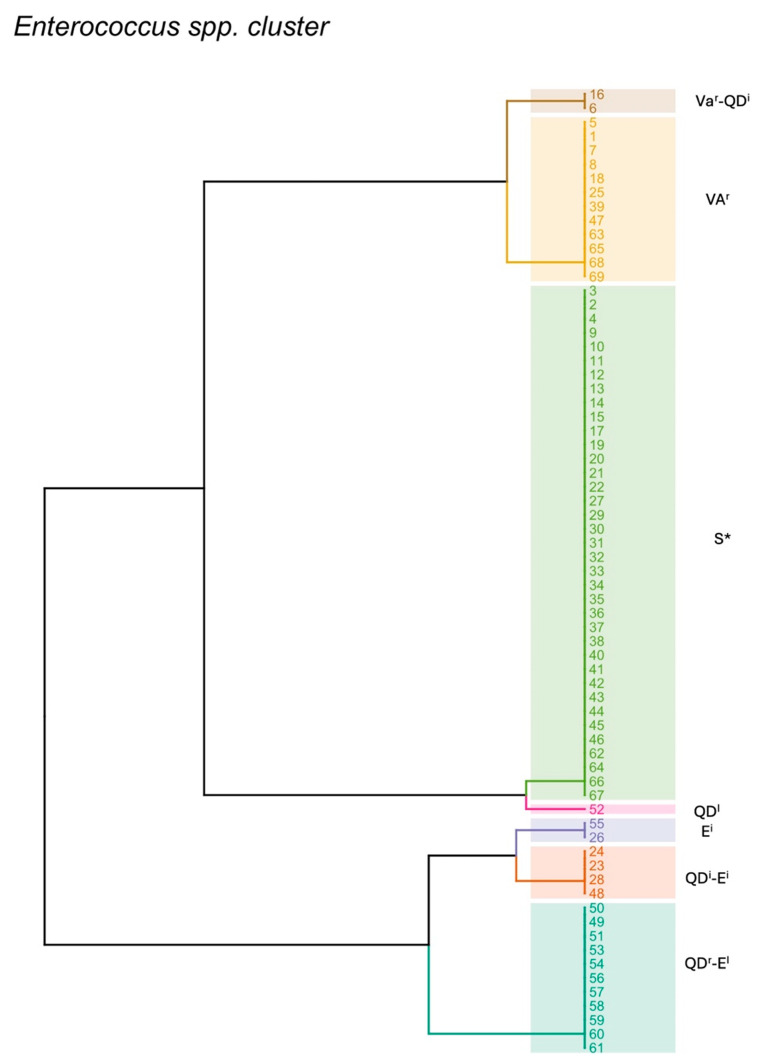
*Enterococcus* spp. clusters based on their phenotypic resistance profiles. Colors indicate different clusters and numbers indicate isolates ID. VA: vancomycin; QD: quinupristin–dalfopristin; E: erythromycin; S*: susceptible to all tested antibiotics; ^r^: resistant; ^i^: intermediate.

Isolates were subsequently grouped based on the different susceptibility profiles obtained. Among 69 isolates, 7 different phenotypic profiles were observed. The most represented profile included isolates susceptible to all tested antibiotics (n = 37), the second most represented profile included isolates showing resistance to vancomycin (n = 12). A third profile included isolates showing resistance to quinupristin–dalfopristin and intermediate resistance to erythromycin (n = 11). A less abundant profile was composed of isolates presenting intermediate resistance to quinupristin–dalfopristin and erythromycin (n = 4). Finally, the remaining three profiles included two or one isolates (Figure 1).

Subsequently, 11 isolates were selected to be subjected to identification through *16S rRNA* gene sequencing, each coming from a profile presenting at least one resistance to one of the tested antibiotics. The selected isolates were attributed to the species *Enterococcus faecalis* (n = 6) and *Enterococcus gallinarum* (n = 5). Table 2 shows the identified species and their respective resistance profiles.

### 3.2. Determination of Antibiotic Resistance Profile and Identification of Enterobacteriaceae

In total, 24 out of the 25 analyzed samples (96%) tested positive for the presence of *Enterobacteriaceae*. From these samples, 62 isolates presumably belonging to the *Enterobacteriaceae* family were obtained. Three samples returned negative results for the presence of *Enterobacteriaceae*. In total, 33.9% of *Enterobacteriaceae* isolates were susceptible to all the tested antimicrobials, 37.1% presented an intermediate resistance to at least one antimicrobial, while 53.2% were found to be resistant to at least one antimicrobial.

The highest rates of resistance were against cefoxitin (37.1%), followed by ampicillin (33.9%), amoxicillin–clavulanic acid (25.8%), aztreonam (4.8%), tetracycline (1.6%), and cefotaxime (1.6%). None of the isolates were resistant to streptomycin, chloramphenicol, imipenem, trimethoprim/sulfamethoxazole, or gentamycin. Resistance rates are shown in Table 1.

**Figure 2:**
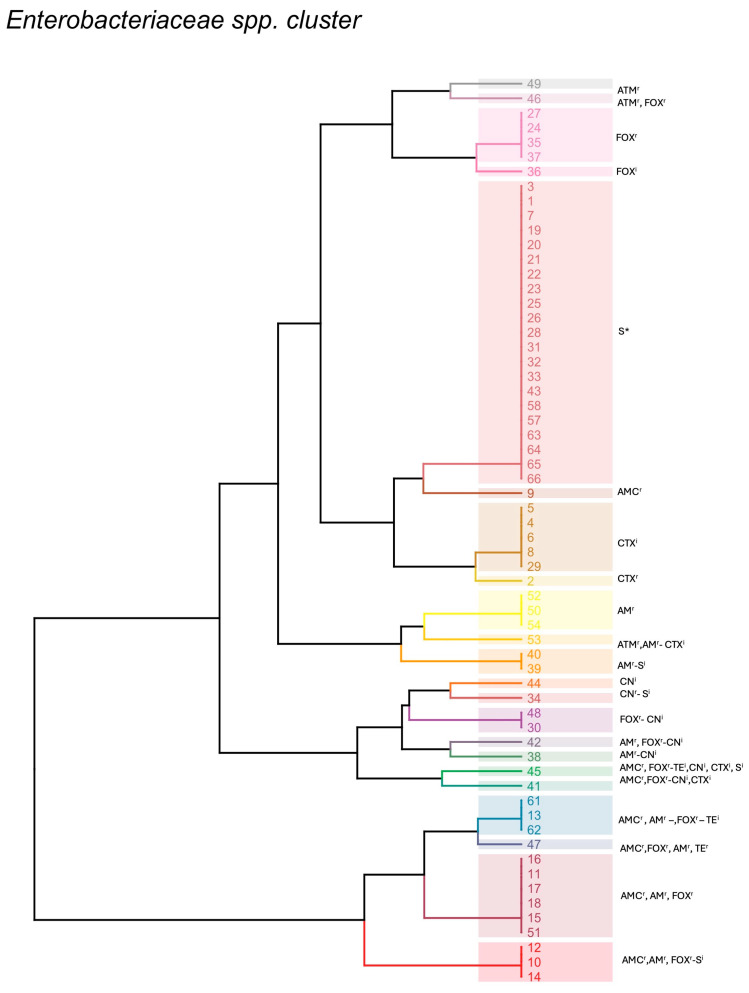
*Enterobacteriaceae* spp. clusters based on their phenotypic resistance profiles. Colors indicate different clusters and numbers indicate isolates ID. E: erythromycin; AMC: amoxicillin–clavulanic acid; AM: ampicillin; FOX: cefoxitin; S: streptomycin; TE: tetracycline; CN: gentamycin; CTX: cefotaxime; ATM: aztreonam; S*: susceptible to all tested antibiotics; ^r^: resistant, ^i^: intermediate.

All isolates were grouped based on their susceptibility profiles, resulting in 22 distinct profiles denoting a higher variability compared with that of the *Enterococcus* spp. The most represented profiles included isolates susceptible to all tested antimicrobials (n = 21); a second profile included isolates resistant to amoxicillin–clavulanic acid, ampicillin, and cefoxitin (n = 5), while the other profiles included isolates resistant to cefoxitin (n = 4), isolates resistant to amoxicillin–clavulanic acid, ampicillin, and cefoxitin and showing intermediate resistance to tetracycline (n = 3), and isolates resistant to amoxicillin–clavulanic acid, ampicillin, and cefoxitin and showing intermediate resistance to streptomycin (n = 3). All the other phenotypic profiles were represented by one or two isolates. Among them, one profile was of particular interest since it was characterized by simultaneous resistance to tetracycline, amoxicillin–clavulanic acid, ampicillin, and cefoxitin (Figure 2).

Subsequently, 19 isolates were selected to be subjected to identification via *16S rDNA* gene sequencing, each coming from a profile presenting at least one resistance to one of the tested antibiotics. The selected isolates were attributed to the species *Enterobacter hormaechei* (n = 6), *Enterobacter bugandensis* (n = 1), *Shigella boydii* (n = 3), *Klebsiella grimontii* (n = 5), *Serratia marcescens* (n = 3), and *Citrobacter amalonaticus* (n = 1). Table 2 shows the identified isolates and their respective resistance profiles.

## 4. Discussion

As reported by other authors [26], all the analyzed *T. molitor* larvae and frass samples were contaminated by enterococci and *Enterobacteriaceae*, which seem to be good indicators for the evaluation of the presence of antibiotic resistant microorganisms. A correct interpretation of the results obtained from the antibiograms cannot ignore the awareness that the different microbial species can present intrinsic resistances that cannot be transferred horizontally and therefore raise minor concern in terms of diffusion.

As for enterococci, we developed some hypotheses on the nature of the resistances observed, trying to distinguish between those of the intrinsic and non-transferable type and those of the acquired type that are therefore transferable to other bacterial species. *E. faecalis* represents a normal host in the gastrointestinal tracts of humans and animals and is one of the etiological agents most implicated in nosocomial infections, including infective endocarditis in particular. In the present study, *E. faecalis* isolates were resistant to quinupristin–dalfopristin and showed intermediate resistance to erythromycin. It is known that unlike *Enterococcus faecium*, *E. faecalis* possesses an intrinsic resistance to quinupristin–dalfopristin [27]. Strains belonging to *Enterococcus* spp. are also naturally resistant against macrolides (erythromycin) due to several chromosomal genes [28]. The other enterococcal species identified in the present study was *E. gallinarum*, which showed resistance to vancomycin. This type of resistance was expected, as all strains belonging to *E. gallinarum* possess *vanC* genes at the chromosomal level which confer resistance to low concentrations of vancomycin [29]. The bacterial wall is made up of peptidoglycan that is formed when wall pentapeptide precursors ending in D-Ala-d-Ala translocate from the cytoplasm to the cell surface. Glycopeptides, such as vancomycin, work through exerting their antibacterial effect via binding with high affinity to the peptidoglycan precursor D-Ala-d-Ala. Glycopeptide resistance occurs when a ligase encoded by the *vanC* gene catalyzes the synthesis of D-Ala-d-Ser and replaces the dipeptide D-Ala-D-Ala in peptidoglycan precursors. This substitution is presumed to reduce the affinity of vancomycin for its target site [30]. Currently, eight phenotypic variants of acquired glycopeptide resistance in enterococci have been described (encoded by the *vanA*, *vanB*, *vanD*, *vanE*, *vanG*, *vanL*, *vanM*, and *vanN* genes), with one type of intrinsic resistance (*vanC*) that is unique to *E. gallinarum* and *Enterococcus casseliflavus* [31].

As for *Enterobacteriaceae*, several resistant isolates were identified belonging to different bacterial genera. The most represented genus was *Enterobacter*, which includes Gram negative, non-spore forming, and facultative anaerobic bacteria. These microorganisms are environmental saprophytes and are also part of the commensal microbiota of the human gastrointestinal tract. *Enterobacter* has also been associated with the natural microbiota of mealworms. A recent study [32] indicated *Enterobacter* spp. as the most abundant OTU (26.2%) in dried mealworm larvae. In addition, other authors [33,34] have described the presence of species belonging to *Enterobacter* spp. in different batches of mealworm larvae and lesser mealworm larvae food preparations (minced meat-like products). In recent decades, *Enterobacter* spp. have assumed clinical significance as many of these species have emerged as human nosocomial pathogens [35]. They belong to the so-called ESKAPE bacterial group, where ESKAPE is an acronym indicating *Enterococcus faecium*, *Staphylococcus aureus*, *Klebsiella pneumoniae*, *Acinetobacter baumannii*, *Pseudomonas aeruginosa*, and *Enterobacter* species. These bacteria are common causes of life-threatening nosocomial infections among immunocompromised patients and are characterized by drug-resistance mechanisms [36]. In the present study, *E. hormaechei* and *E. bugandensis* were detected. *E. hormaechei* belongs to the “*Enterobacter cloacae* complex” together with *Enterobacter cloacae*, *Enterobacter asburiae*, *Enterobacter kobei*, *Enterobacter ludwigii*, and *Enterobacter nimipressuralis*. From a phylogenetic point of view, *E. bugandensis* is very similar to *E. hormaechei*. *E. bugandensis* was described for the first time in 2011 in Tanzania and was associated with neonatal sepsis [37]. In the present study, both *E. hormaechei* and *E. bugandensis* showed resistance to some beta-lactam antibiotics and their association with beta-lactamase inhibitors (ampicillin, cefoxitin, and amoxicillin–clavulanic acid) and were instead susceptible to third-generation cephalosporins (ceftazidime) and carbapenems (imipenem). Strains belonging to the *E. cloacae* complex are able to produce a constitutive AmpC-type beta-lactamase [35]; therefore, the resistances observed for *E. hormaechei* seem to be intrinsic. Considering the phylogenetic closeness between *E*. *hormaechei* and *E. bugandensis*, it is reasonable to suspect that this is also the case for the resistances observed for *E. bugandensis* [35]. Various species of enterobacteria produce beta-lactamases due to the presence of genes located on the chromosome. AmpC type beta-lactamases are active against penicillins, first generation cephalosporins, and cephamycins (cefoxitin). These beta-lactamases are not inhibited by clavulanic acid or penicillinic acid sulfones. However, some strains also possess genes encoding AmpC-type beta-lactamases on plasmids [35]. These strains are easily identifiable, because generally, unlike what happens in strains harboring *ampC* at the chromosomal level, the production of lactamase is not inducible. In this case, only penicillins, narrow spectrum cephalosporins, and cephamycins function as inducers. This explains why species belonging to the *E. cloacae* complex are intrinsically resistant to the latter antibiotics, but not to third-generation cephalosporins. On the contrary, strains possessing the *ampC* gene at the plasmid level are also resistant to third-generation cephalosporins [38].

*Serratia* spp. also include species considered for a long time to be environmental and non-pathogenic [39]. In this study, three isolates belonging to *S. marcescens* were identified. This species is among the most studied *Serratia* species since it is frequently associated with infections such as pneumonia, septicemia, necrotizing fasciitis, and various nosocomial infections. Recently, *S. marcescens* was identified as an opportunistic entomopathogen and, due to its relatively low virulence and the persistence in the environment, it was proposed as an indicator for the sanitary status of mealworm production [40].

*S. marcescens* isolates from this study showed resistance against ampicillin, amoxicillin-clavulanic acid, and cefoxitin. These observed resistances were not unexpected and are explainable through *Serratia* species’ intrinsic resistance to beta-lactams. Indeed, the production of chromosomally encoded AmpC-type beta-lactamases in *Serratia* spp. isolates, as well as in *S. marcescens*, has been well documented [41]. Similar to what has been observed for the *E. cloacae* complex, this enzyme confers resistance to penicillins, first-generation cephalosporins, and cephamycins and is not inhibited by clavulanic acid. Resistance to third-generation cephalosporins and aztreonam can occur if the *ampC* gene is overexpressed [39].

It is interesting to note that among the three *S. marcescens* isolates, two were intermediate resistant to tetracycline and one was tetracycline-resistant. Indeed, *S. marcescens* should express intrinsic resistance to tetracycline due to the presence of the *tetA* gene encoding an MFS efflux pump [42]. Isolate 45 also showed intermediate resistance to cefotaxime, gentamicin, and streptomycin. Concerning aminoglycosides, genes coding for resistance such as *aph*(*3″*) and *aac*(*6′*)*-Ic* have been identified on many chromosomes of strains belonging to the *Serratia* genus [43].

Among resistant isolates, we were able to identify five *K. grimontii*. The *Klebsiella* genus includes opportunistic pathogenic species that affect both humans and animals. Several studies reported the presence of *Klebsiella* spp. in mealworm larvae and samples of dried ready-to-eat mealworms [26,33]. In humans, bacteria belonging to this genus are implicated in various pathologies of the gastrointestinal tract including antibiotic-associated colitis and nosocomial infections caused mainly by ESBL-producing strains [44,45]. In the present work, five *K. grimontii* isolates showed resistance against some beta lactams, such as ampicillin, cefoxitin, and aztreonam, with different phenotypic profiles. In the work of Passet and Brisses (2018) [46], *K. grimontii* was described for the first time as very similar from a phylogenetic point of view to *Klebsiella oxytoca*. This microorganism, as well as *Klebsiella pneumoniae*, due to the presence of chromosomal genes, produces class A beta-lactamases, SHV-1, and K1, respectively, conferring the ability to hydrolyze penicillins and narrow-spectrum cephalosporins. Furthermore, some strains of *K. oxytoca* can overproduce the K1 enzyme, resulting in a resistance towards aztreonam [38]. This resistance was observed in *K. grimontii* isolates from the present study.

Interesting results were obtained for *Shigella boydii* isolates. *Shigella* spp. are responsible for shigellosis in humans. The bacteria belonging to this genus invade the mucosa of the colon, causing diarrheal phenomena up to the hemolytic–uremic syndrome in those most at risk, such as children [47]. *Shigella* spp. do not possess intrinsic resistance to β-lactams. However, the CLSI standards (for isolates of human and veterinary origin) report that for *Salmonella* spp. and *Shigella* species, both first- and second-generation cephalosporins and cephamycins may appear active in vitro but are not clinically effective and should not be reported as sensitive. In our work, isolates belonging to *S. boydii* were found to be resistant to cefoxitin, a cephamycin belonging to the class of beta-lactam antibiotics, and in one case to aztreonam (beta-lactam belonging to the class of monobactams). Evidence for the presence of a constitutive resistance towards cephalosporins or monobactams in *S. boydii* is not present in the literature. Thus, further investigations will be necessary to identify the genetic nature of the observed resistance phenotypes.

Lastly, one isolate belonging to *Citrobacter amalonaticus* species was identified. *Citrobacter* can represent an opportunistic pathogen for immunocompromised patients. The identified isolate showed resistance against ampicillin, amoxicillin–clavulanic acid, and cefoxitin. Chromosomal inducible AmpC beta-lactamases in this bacterial population have been well described and represent an important mechanism of resistance to beta-lactams [48]. Again, for this species, the observed results can be explained as being due to intrinsic resistance.

## 5. Conclusions

The presence of antibiotic-resistant bacteria in edible insects necessitates consideration when assessing their food safety. A thorough evaluation of the risk associated with insect consumption is warranted due to the increasing interest in their utilization as an alternative protein source for both human and animal diets. Although insects are already incorporated into food and livestock feed in numerous regions globally, their adoption remains a topic of debate. The primary barrier to the trade and utilization of edible insects stems from a lack of understanding regarding their potential as carriers of chemical and microbiological hazards. Recent research has characterized the bacterial communities within the microbiota of key edible insects, yet the available data are not sufficient. Specifically, investigations into the potential transmission of genes encoding antibiotic resistance by these insects have been limited. The presence of antibiotic-resistant pathogens in animal-derived food products poses a significant risk to human health. The extensive and inappropriate use of antibiotics in human and veterinary medicine, as well as in livestock farming and agriculture, has exerted significant selective pressure over time, resulting in a dramatic increase in antibiotic-resistant bacterial strains, including those responsible for serious infections in humans [49,50]. The genes responsible for antibiotic resistance can easily be transferred through horizontal transmission among different bacterial communities, and insects may also serve as a potential means of dissemination [22]. In accordance with international food standards, the presence of antibiotic-resistance determinants must be considered in the risk analysis of food products. Given this, and considering the large-scale breeding of insects and the potential for bacterial infections that could affect these animals, it is important to understand their intestinal microbiota and the potential abundance of resistant strains that could be selected and predominate in the event of antibiotic treatments.

The data presented in this study originated from insects reared in a laboratory setting under controlled conditions. In summary, within the specific experimental conditions, the observed resistance profiles of the selected microorganisms were due to intrinsic mechanisms characteristic of the identified species. Thus, the presence of transmissible resistance genes was not hypothesized. It is important to acknowledge the limitations of this study, primarily related to the culture-dependent methodology utilized, which provides information solely on viable and cultivable target microorganisms, thus not ruling out the potential existence of other resistant microbial species.

Future research efforts should prioritize the exploration of sources and factors contributing to the dissemination of resistance determinants within the large-scale breeding and production chains of edible insects. Additionally, it will be valuable to assess the evolution of bacterial communities in relation to farming conditions, dietary regimens, therapeutic interventions, and throughout the stages of processing, storage, and transportation, employing an integrated approach that combines culture-dependent and -independent methodologies. In conclusion, numerous challenges remain to be addressed concerning the production, processing, and marketing of a product that holds the potential for increased prevalence across various countries in the future.

## Figures and Tables

**Table 1:** Numbers and percentages of isolates resistant against different antibiotics employed in human and veterinary medicine.

	Antibiotics	% Resistant Isolates (n)	% Intermediate Isolates (n)
*Enterococcus*	Vancomycin	20.3 (14)	0
	Quinupristin–Dalfopristin	15.9 (11)	10.1 (7)
	Erythromycin	0	24.6 (17)
*Enterobacteriaceae*	Cefoxitin	37.1 (23)	1.6 (1)
	Ampicillin	33.9 (21)	0 (0)
	Amoxicillin–Clavulanic acid	25.8 (16)	0 (0)
	Aztreonam	4.8 (3)	
	Tetracycline	1.6 (1)	6.4 (4)
	Cefotaxime	1.6 (1)	12.9 (8)
	Streptomycin	0 (0)	11.3 (7)
	Gentamycin	0 (0)	12.9 (8)

**Table 2:** List of identified isolates with their phenotypic resistance profiles.

	Isolate ID	Species	Phenotypic Profile
*Enterococcus*	1	*Enterococcus gallinarum*	VA^r^
	5	*Enterococcus gallinarum*	VA^r^
	6	*Enterococcus gallinarum*	VA^r^-QD^i^
	8	*Enterococcus gallinarum*	VA^r^
	50	*Enterococcus faecalis*	QD^r^-E^i^
	51	*Enterococcus faecalis*	QD^r^-E^i^
	56	*Enterococcus faecalis*	QD^r^-E^i^
	57	*Enterococcus faecalis*	QD^r^-E^i^
	60	*Enterococcus faecalis*	QD^r^-E^i^
	61	*Enterococcus faecalis*	QD^r^-E^i^
	68	*Enterococcus gallinarum*	VA^r^
*Enterobacteriaceae*	10	*Enterobacter hormaechei*	AMC^r^, AM^r^, FOX^r^-S^i^
	12	*Enterobacter hormaechei*	AMC^r^, AM^r^, FOX^r^-S^i^
	16	*Enterobacter bugandensis*	AMC^r^, AM^r^, FOX^r^
	13	*Enterobacter hormaechei*	AMC^r^, AM^r^, FOX^r^-TE^i^
	14	*Enterobacter hormaechei*	AMC^r^, AM^r^, FOX^r^-S^i^
	30	*Shigella boydii*	FOX^r^-CN^i^
	35	*Shigella boydii*	FOX^r^
	38	*Klebsiella grimontii*	AM^r^-CN^i^
	41	*Enterobacter hormaechei*	AMC^r^, FOX^r^-CN^i^, CTX^i^
	45	*Serratia marcescens*	AMC^r^, FOX^r^-TE^i^, CN^i^, CTX^i^, S^i^
	46	*Klebsiella grimontii*	ATM^r^, FOX^r^
	39	*Klebsiella grimontii*	AM^r^-S^i^
	42	*Klebsiella grimontii*	AM^r^, FOX^r^-CN^i^
	47	*Serratia marcescens*	AMC^r^, FOX^r^, AM^r^, TE^r^
	49	*Shigella boydii*	ATM^r^
	50	*Enterobacter hormaechei*	AM^r^
	51	*Citrobacter amalonaticus*	AMC^r^, AM^r^, FOX^r^
	53	*Klebsiella grimontii*	ATM^r^, AM^r^-CTX^i^
	61	*Serratia marcescens*	AMC^r^, AM^r^, FOX^r^-TE^i^

VA: vancomycin; QD: quinupristin–dalfopristin; E: erythromycin; AMC: amoxicillin–clavulanic acid; AM: ampicillin; FOX: cefoxitin; S: streptomycin; TE: tetracycline; CN: gentamycin; CTX: cefotaxime; ATM: aztreonam; ^r^: resistant; ^i^: intermediate.

## Data Availability

The data presented in this study are available on request from the corresponding author.
